# Survey on 5G Physical Layer Security Threats and Countermeasures

**DOI:** 10.3390/s24175523

**Published:** 2024-08-26

**Authors:** Michal Harvanek, Jan Bolcek, Jan Kufa, Ladislav Polak, Marek Simka, Roman Marsalek

**Affiliations:** Department of Radio Electronics, Faculty of Electrical Engineering and Communication, Brno University of Technology, Technicka 3082/12, 616 00 Brno, Czech Republic; michal.harvanek@vut.cz (M.H.); 183892@vutbr.cz (J.B.); kufa@vutbr.cz (J.K.); polakl@vut.cz (L.P.); xsimka01@vut.cz (M.S.)

**Keywords:** 4G, 5G, security vulnerabilities, Physical Layer (PHY), machine learning, eavesdropping, jamming, spoofing, localization

## Abstract

With the expansion of wireless mobile networks into both the daily lives of individuals as well as into the widely developing market of connected devices, communication is an increasingly attractive target for attackers. As the complexity of mobile cellular systems grows and the respective countermeasures are implemented to secure data transmissions, the attacks have become increasingly sophisticated on the one hand, but at the same time the system complexity can open up expanded opportunities for security and privacy breaches. After an in-depth summary of possible entry points to attacks to mobile networks, this paper first briefly reviews the basic principles of the physical layer implementation of 4G/5G systems, then gives an overview of possible attacks from a physical layer perspective. It also provides an overview of the software frameworks and hardware tool-software defined radios currently in use for experimenting with 4G/5G mobile networks, and it discusses their basic capabilities. In the final part, the paper summarizes the currently most promising families of techniques to detect illegitimate base stations—the machine-learning-based, localization-based, and behavior-based methods.

## 1. Introduction

With the recent developments of mobile networks, users profit from ubiquitous connectivity, ever-increasing data rates, and a wide range of emerging applications. On the other hand, our dependence on communication technology can lead to increased, or completely new, security risks. Cellular networks such as the Fourth Generation (4G) and Fifth Generation (5G) systems of mobile communications are among the most widespread and commonly used communication systems nowadays. As such, they are often used for managing various private systems as well as critical infrastructure, making them potential and attractive targets for cyberattacks [1,2]. Attacks on mobile networks usually aim to compromise at least one of the requirements of secure communication, such as confidentiality, integrity, accountability, availability, or privacy. On top of that, several unprecedented risks may arise from the use of currently very popular Open Radio Access Network (RAN) architecture, as mentioned in the current report of the German Federal Office for Information Security on security related to Open RAN [3].

In the last decade, several studies of 4G and 5G cellular networks have dealt with their security risks, e.g., [4] they have focused on the threats to voice and short message services. The survey paper [5] presented techniques for physical layer authentication, but only from the general methodological perspective. Study [6] was aimed only at the deep-learning techniques for physical layer security themselves. In contrast to [2], we include up-to-date 5G-related findings in the domain of physical layer security, provide an overview of machine learning, localization, and behavior-based methods to detect malicious base stations and include an overview of hardware tools and open RAN architectures suitable for practical experimentation.

**Figure 1:**
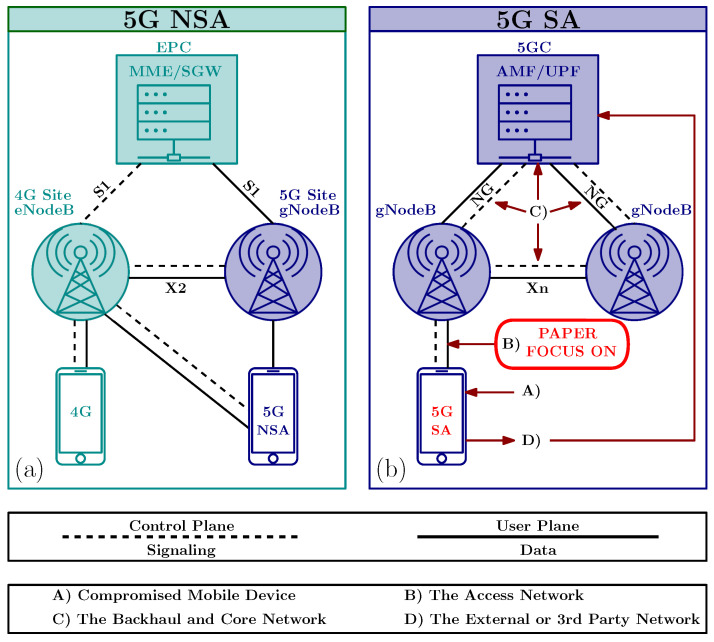
(**a**) SA vs. (**b**) NSA architecture of 5G New Radio (NR) network with possible vector attacks.

Although the focus of this study is on the techniques related to the Physical (PHY) layer, prior to diving deep into PHY and corresponding threats, we first provide an overview of the possible attack entry points. In Figure 1, the topology of a 4G and 5G network defines possible attack vectors for network breaches. While both Non Stand Alone (NSA) and Stand Alone (SA) architectures aim to deliver the benefits of 5G, they differ significantly in their approach to network infrastructure and security. The 5G NSA networks leverage existing 4G Long Term Evolution (LTE) infrastructure, sharing the 4G core network Evolved Packet Core (EPC) for the control plane, with the 5G RAN providing enhanced user plane capabilities. In contrast, 5G SA networks are built from the ground up, with all functions, including the control and user planes, residing in the 5G Core Network (5GC). Depending on the network architecture, whether its 4G, 5G NSA, or 5G SA, potential entry points for threats can vary. These entry points can generally be categorized into four groups: the compromised mobile device, the access network, the backhaul network, and external or third-party networks, where each category can implement various technologies.

### 1.1. Entry Points for Attacks to 5G Networks

#### 1.1.1. Compromised Mobile Device

The first possible entry point is compromised or malicious User Equipment (UE). This is a significant threat to mobile networks, serving as both targets and enablers for attacks. Malware spread through application downloads is a common method of compromising devices [7,8].

Compromised device attacks are also influenced by the behavior of UE users. Risky actions, such as downloading from unofficial application stores [9], connecting to unknown Bluetooth [10] and WiFi [11] devices, scanning harmful QR codes (phishing), or receiving malicious messages via SMS or communication apps [12,13], can lead to attacks on mobile devices. Mobile botnets, facilitated by malware like Trojan horses, are emerging as major threats, allowing the remote control of compromised devices. Denial of Service (DoS) or Distributed Denial of Service (DDoS) attacks on mobile networks can be launched through malware on user equipment, targeting specific network components [14,15]. Furthermore, malicious applications can compromise devices through permission grants, and threats from the internet, phishing, and Man-In-The-Middle (MITM) attacks also pose risks.

#### 1.1.2. The Access Network

The second entry point can reside in the access network, particularly concerning the S1/NG interface in 4G/5G (see Figure 1) and this poses significant risks to mobile networks. Vulnerabilities related to the access network include using gNodeBs (gNBs) to attack the core service and inject fake traffic into applications. Weaknesses in data and signaling encryption further exacerbate these risks. During initial authentication procedures, the messages exchanged lack encryption and integrity protection, potentially exposing sensitive data like International Mobile Subscriber Identity (IMSI) [16]. The absence of authentication between Serving and Home Networks, along with inadequate cryptosystems, leaves networks vulnerable to redirection attacks and message modification. Seamless interoperation between access technologies, such as Global System for Mobile communications (GSM), Universal Mobile Telecommunications System (UMTS), and LTE, also presents security challenges [17,18,19], especially with the combination of downgrade attacks, as reported in [20]. The GSM Authentication and Key Agreement (AKA) protocol suffers from weaknesses, including a lack of mutual authentication and integrity protection, which can be exploited [17]. The storage of authentication triplets in the Visitor Location Register (VLR) further exposes vulnerabilities [17]. Additionally, signaling overload, limited bandwidth, and heavy processing in Radio Resource Control (RRC) procedures create opportunities for attacks in the Core Network (CN) [21].

#### 1.1.3. The Backhaul and Core Network

The third entry point includes the backhaul and CN [22,23]. The backhaul, which is responsible for data transfer between the RAN and the CN, presents a potential access point for attackers to intercept control and data traffic. Integration of diverse access technologies like femtocells and non-Third Generation Partnership Project (3GPP) WiFi [2] introduces new vulnerabilities, particularly in 4G/5G, with interfaces like X2/Xn (see Figure 1) and diameter signaling protocols amplifying signaling overload and transitioning to Internet Protocol (IP). The EPC CN faces severe threats due to its flat IP-based architecture and direct connections from Base Stations (BSs) to the ALL-IP network [22]. Weaknesses in the Evolved Packet System (EPS)-AKA scheme, handover procedure, and Machine Type Communication (MTC) security architecture exacerbate risks [22,24]. Additionally, vulnerabilities in the General Packet Radio Service (GPRS) Tunnelling Protocol (GTP) protocol used in EPC Non-Access Stratum (NAS) expose networks to abnormal packet threats and traffic analysis [25]. Furthermore, virtualization and software-defined networks in the CN introduce emerging threats by dispersing user and control-plane traffic across network elements and non-trusted networks [7].

#### 1.1.4. The External or 3rd Party Network

An external or 3rd party network serves as a fourth entry point for threats against mobile networks, offering various user services, including internet browsing, corporate network interconnection, and roaming partner networks. Non-3GPP access networks, especially when interworking with a Wireless Local Area Network (WLAN) [2], introduce security issues, such as disclosure of user information, permanent identity tracking, and network impersonation [26]. Additionally, bypassing access control and authentication processes, interference with charging, and prevention of user access to services are concerns [26,27,28]. Converged networks utilizing technologies like Worldwide Interoperability for Microwave Access (WiMAX) also pose threats due to weaknesses in the physical and Multiple Access Channel (MAC) layers, thereby leaving them vulnerable to spoofing, MITM, and eavesdropping attacks [29].

### 1.2. General Countermeasures for the Access Network, Backhaul, and Core Network

To protect the 5G network, 3GPP has established specific and detailed security objectives [30]. These objectives are closely aligned with the 5G security architecture and procedures, which are divided into five key areas [31]:

- Confidentiality: Ensures only authorized users access confidential data.
- Integrity: Protects against unauthorized data modification, ensuring data is transmitted in its original form.
- Authentication: Verifies the identities of entities (UEs, network functions, serving networks, Public Land Mobile Networks (PLMNs)) before communication, which involves a key exchange.
- Replay Protection: Prevents attackers from capturing and reusing packets for illegitimate communication.
- Privacy: Protects users’ sensitive data, including inferred information like habits, profiles, and location, from unauthorized access.

**Figure 2:**
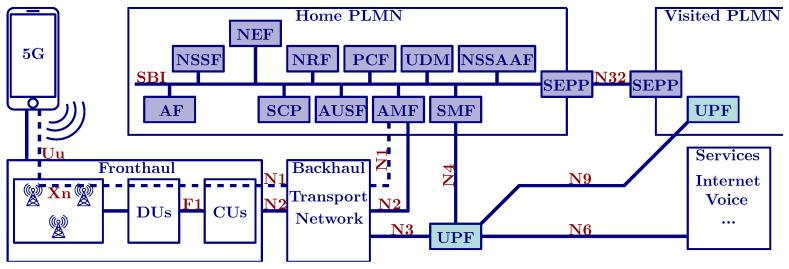
5G architecture block scheme with critical interfaces (red text) and its end points. Purple and blue colors represent Control Plane (CP) and User Plane (UP) functions respectively.

Authors in [1,32] surveyed the security requirements recommended by 3GPP for Device-to-Device (D2D), Internet of Things (IoT), Vehicle-to-Everything (V2X), network slicing, network function virtualization, mobile edge computing, and other 5G-specific technologies. The authors in [33] highlighted weaknesses, such as a scenario where an attacker, by reusing a previous key, could force the UE and serving network, establishing a secure communication link. This could potentially enable the reply of user data. To cope with this issue, they also suggested a protocol improvement to use a different key for each session. They also confirmed that IMSI catchers are defeated by the use of a randomized public key encryption. The authors in [34] provided a performance overhead comparison for various optional AKA integrity protection algorithms applied on the user plane. Practically, the throughput and latency are minimally affected, while the security of the network is increased. In [35] the authors analyzed critical 5G interfaces and their endpoints, as depicted in Figure 2. They provided suggested improvements for system interfaces, which can be summarized by the following points:

- N1 and Uu Interfaces: Ensuring the integrity of the control plane on the N1 and Uu interfaces, as well as the Permanent Equipment Identifier (PEI) on N1, is crucial and is enforced using NIA1-Network Integrity Algorithms (NIA)3. Operators have the discretion to choose the level of confidentiality using Network Encryption Algorithms (NEA)1-NEA based on their specific requirements. The Subscriber Permanent Identifier (SUPI) and the Subscriber Concealed Identifier (SUCI) should be protected using the Elliptic Curve Integrated Encryption Scheme (ECIES). Additionally, operators can determine the integrity and confidentiality measures for the user plane on the Uu interface by selecting the appropriate NIA1-NIA3 and NEA1-NEA3 algorithms.
- N2, N3, Xn, and F1 Interfaces: To safeguard confidentiality and integrity and protect against replay attacks on the N2, N3, Xn, and F1 interfaces, operators can implement IP Security (IPsec) Encapsulating Security Payload (ESP), along with Internet Key Exchange version 2 (IKEv2) utilizing certificate-based authentication.
- Service-Based Interface (SBI) Interface: To ensure confidentiality, integrity, and protection against replay attacks on the SBI, operators can employ Transport Layer Security (TLS). This protocol encrypts data, verifies its integrity, and prevents unauthorized retransmissions, ensuring a secure communication channel for the SBI interface.

Note that the above presented security mechanisms involve rather higher layers than PHY, but they are worth mentioning for the sake of completeness.

### 1.3. Paper Structure and Organization

Traditional cryptography solutions, while crucial, cannot address all security challenges in 5G networks, not only because of their inherent complexity and computational demands but primarily because many attacks happen in the initial phase of the connection, when communication is usually unprotected. This paper explores the role of PHY layer security in 5G and its potential to safeguard communications against evolving threats. Therefore, we focus primarily on the vulnerabilities of the lower layers, such as the PHY layer, and on the potential use of the PHY for authenticating legitimate communication devices. This article is divided as follows: In the Section 2, we review the technology background of the 5G PHY layer. In Section 3, we focus on possible basic threats to mobile networks such a jamming, smart jamming, and spoofing/overshadowing attacks, and we complement it with the basic countermeasure approaches. Section 4 is focused on Multiple-Input Multiple-Output (MIMO)-specific attacks such a Beam Alignment (BA) jamming, pilot contamination, and user tracking, together with the most common mitigation techniques. Hardware and open-source software tools, which can be used to build 4G/5G testbeds for experimentation, are introduced and thoroughly compared in Section 5. Section 6, Section 7, and Section 8, respectively, present surveys of machine-learning techniques and methods that rely on localization information and device behavior to detect the most common threat—the rogue base station. Section 9 then discusses the PHY layer security related to the future technology candidates for beyond 5G networks. Finally, Section 10 concludes the paper.

## 2. Technology Background of Physical Layer

The PHY layer of wireless communication systems is responsible for transmitting and receiving data over the air interface. The PHY is also a potential target of various security attacks, such as jamming, spoofing, eavesdropping, and relay attacks. These attacks can degrade the performance, reliability, and confidentiality of wireless communication. Therefore, it is important to design and implement security mechanisms at the PHY level, in addition to at the higher layers of the protocol stack.

### 2.1. Frequency Bands in 5G

The 5G standard is a revolutionary step in the field of wireless communication, utilizing Frequency Range 1, Frequency Range 2, and New Radio Unlicensed (NR-U) to cater to a wide array of requirements and scenarios. In the following, we provide a summary of 5G NR frequency bands.

#### 2.1.1. 5G FR1 Sub-6 GHz Band

The Frequency Range 1 (FR1) encompasses the sub-6 GHz spectrum, some of which is used by previous standards. Higher-order MIMO in FR1 bands enables spatial multiplexing and Multi User Multiple-Input Multiple-Output (MU-MIMO), offering a balance of coverage and capacity, making it suitable for wide-area deployments and ensuring compatibility with existing 4G networks. FR1 frequency bands offer a balance between coverage and capacity. They provide wider coverage compared to higher frequency bands such as those in Frequency Range 2 (FR2), or millimeter-wave bands, typically used in urban, suburban, and rural areas. Additionally, FR1 bands offer better penetration through buildings and obstacles, enhancing indoor coverage.

#### 2.1.2. 5G FR2 mmWave Band

The FR2, on the other hand, utilizes mmWaves and refers to frequency bands above 24.25 GHz and up to 52.6 GHz in order to achieve low latency and wide bandwidth to achieve high data throughput. This frequency band is typically used in small femto-cells to cover small areas, typically building floors.

#### 2.1.3. 5G Spectrum Expansion to FR3 FR4 FR5 Bands

There is ongoing discussion [36,37] aimed at utilizing other frequency bands in 5G. The current hot topic is a discussion about the use of frequency ranges FR3, FR4, and FR5. Basically, the plan is to reserve frequencies ranging from 7.125 GHz to 24.25 GHz, and from 52.6 GHz to 71 GHz for future releases of 5G. Each manufacturer (Qualcomm, Apple, Lenovo, Nokia, …) pick different names for each range. Some manufacturers use the FR3 label for range 7.125 GHz to 24.25 GHz, and some manufacturers use FR3 naming for the frequency range 52.6–71 GHz. However, regardless of the names, it is likely that the mentioned frequency bands will be used in the future.

#### 2.1.4. 5G New Radio Unlicensed Band

The 5G standard also defines a non-licensed mode named 5G New Radio Unlicensed (NR-U). This mode of operation has been studied in [38] and introduced as a part of the 3GPP Release 16 specifications [39]. It is an evolution of the 4G LTE License Assisted Access (LAA) standards. NR-U provides the necessary technology for cellular operations to integrate an unlicensed spectrum into 5G networks. The RAN provides support for a sidelink in the unlicensed spectrum, specifically aimed at FR1 unlicensed bands (n46 and n96/n102), which are the 5 GHz and 6 GHz [40] unlicensed bands, in line with the most recent regulations [36]. The NR-U is also considered to utilize mmWave frequency ranging from 57 GHz to 71 GHz [41].

There are two operation modes defined for 5G NR-U:

- Anchored NR-U: This requires an anchor in the licensed or shared spectrum. It combines the unlicensed spectrum with the licensed spectrum or shared spectrum such as Citizens Broadband Radio Service (CBRS] to boost deployments for a better user experience with higher 5G speeds.
- Standalone NR-U: This utilizes only the unlicensed spectrum, i.e., it does not require any licensed spectrum. It allows the deployment of 5G private networks entirely with the unlicensed spectrum.

#### 2.1.5. 5G IoT Technologies

The advent of 5G technology has brought significant advancements, not only in the field of wireless personal communication but also in the domain of connected devices—IoT. IoT-based communication and related technologies, characterized by low hardware and operational cost, low power and data rate, and long range communication capabilities, have become integral into many sectors, including industry and healthcare [42]. Critical components of 5G, such as LTE Cat-M and Narrow Band IoT (NB-IoT) technologies, address the growing demand for IoT applications. These technologies are specifically designed for Low-Power Wide Area Network (LPWAN) communications [43], making them ideal for connecting a vast number of IoT devices.

However, these advancements also introduce new security challenges, particularly at the PHY layer of communication [44]. Ensuring robust security at this layer is crucial, as it serves as the foundation for all higher-level security mechanisms. Currently, numerous studies are focusing on the security threats of IoT technologies. Shian et al. [45], in their survey study, examined the Cellular IoT Service Security issues and challenges. They noted that integrating cellular IoT into existing cellular networks can lead to security vulnerabilities due to various operational differences between the cellular IoT and non-IoT devices. Jia et al. [46] addressed the issue of terminal identity trustworthiness in IoT-based mobile communication, specifically targeting forgery attacks in single-package authorization and proposing a solution to handle this problem. Other studies [47,48,49] have explored IoT security issues and their detection using Machine Learning (ML) and Deep Learning (DEL) approaches. In [47], an ML-based IoT intrusion detection model to enhance data processing security and attack detection accuracy was developed. Survey papers [50,51,52] provide overviews of IoT technologies, including LTE Cat-M and NB-IoT, discussing security and vulnerability issues. Addressing these security threats is essential to protect the integrity, confidentiality, and availability of 5G networks [53], ensuring safe and reliable communication for IoT devices.

### 2.2. Parameters and Basic Structure of 5G PHY Layer

Understanding key parameters and PHY layer access is crucial to assess network security. These parameters define aspects like spectrum allocation and initial access procedures, and they have influence on security challenges such as vulnerability to interference or eavesdropping.

#### 2.2.1. 5G Numerology over Frequency Bands

In the realm of NR FR1, the maximum bandwidth is 100 MHz, whereas in the millimeter wave range FR2, it extends up to 400 MHz; see Table 1. Specific Sub-Carrier Spacing (SCS), such as 15 and 30 kHz, are exclusive to the sub 6 GHz range, while 120, 480, and 960 kHz spacing is solely applicable to the millimeter wave range FR2. However, a spacing of 60 kHz can be utilized in both the sub 6 GHz FR1 and the millimeter wave range FR2. The configuration of specific parameters is determined by the network operator. Overview tables of individual channels are provided in references [54,55].

#### 2.2.2. PHY Layer Frame Structure

Depending on whether the deployment type of the 5G network is NSA or SA, the initial access to the network is provided through either 4G or 5G physical channels.

5G technology offers not only faster data rates and lower latency but also a new, flexible frame structure to accommodate a wide range of devices and applications. This structure is more adaptable than its LTE predecessors. It optimizes network utilization by dividing time–frequency resources into blocks and grid elements, ensuring efficient connectivity for various scenarios. The 5G technology implements the so-called time–frequency resource allocation method. The time–frequency resources are divided into resource blocks and further subdivided into resource grid elements.

The fundamental scheduling unit in 5G has shifted from a subframe in 4G to a slot [57]. This allows dynamic adjustment of the time slot duration based on the service type. This flexibility enhances network efficiency and responsiveness. Additionally, the concept of mini-slots provides faster response times for certain applications, which are crucial in emergency situations.

**Figure 3:**
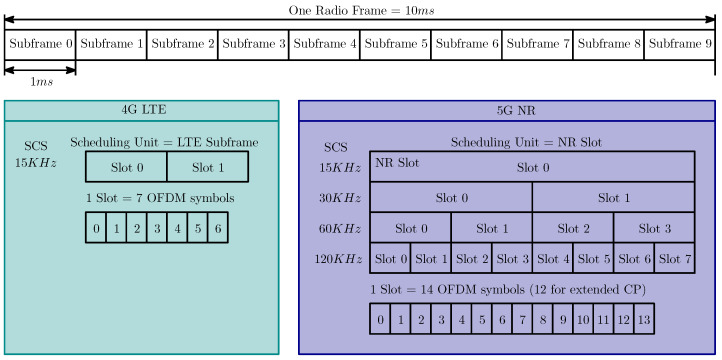
Frame structure comparison between 4G LTE and 5G NR.

The 5G frame structure incorporates both self-contained and non-self-contained subframes, adding another layer of flexibility. This allows for different data transmission methods depending on the application requirements, maintaining service quality and further reducing latency. The 5G radio frame is 10 ms long and, the same as for the 4G, it is divided into 10 subframes with 1 ms duration. Each subframe is divided into slots, depending on which SCS is considered. Each slot contains 14 Orthogonal Frequency Division Multiplexing (OFDM) symbols in one Resource Block (RB) or 12 symbols for the extended CP. With increased SCS, the number of slots in the subframe increases because of the shorter symbol duration (from the OFDM theory, the OFDM symbol duration is inversely proportional to the subcarrier spacing). A comparison of the frame structures for 4G and 5G is depicted in Figure 3.

For the duplexing, it is important that any cellular communication systems must be able to transmit in both directions—Uplink (UL) and Downlink (DL)—simultaneously. To provide the highest possible flexibility, 5G supports various duplexing schemes, such as Frequency Division Duplex (FDD),Time Division Duplex (TDD), Semi-static TDD, and Dynamic TDD. TDD operation is set to be the primary duplex arrangement for higher frequencies in 5G, while lower frequencies continues to utilize FDD. This choice is due to the mitigation of interference issues in larger cells, which can be achieved by employing distinct frequencies for the UL and DL directions.

Frequency Division Duplex refers to a paired spectrum with separate UL and DL carriers. This allows simultaneous data transfer in both directions due to distinct carrier frequencies. Resource allocation is dynamic and independent for each direction, utilizing paired bands. There are two possibilities in FDD. Half duplex mode is used for frequency bands where it is not possible to have simultaneous transmission and reception in both the UL and DL within the cell. It allows simplified device implementation due to the relaxation or absence of duplex filters. For a certain frequency band, it is possible to have simultaneous transmission and reception in both the UL and DL within a cell. One of the drawbacks of this scheme is that the band definition requires a guard band between the UL and the DL, and the receiver must be equipped with a duplex filter to suppress interference from the transmitter.

In the Time Division Duplex, as a type of half duplex scheme, a single carrier frequency is utilized for both the UL and the DL, and their separation is achieved by using different time slots. An essential aspect of any half-duplex system in general is the possibility of providing a sufficiently large Guard Period (GP) or Guard Time (GT), where neither DL nor UL transmissions occur [58]. The length of GT typically increases for stations that cover larger areas. TDD uses unpaired bands, where the UL and DL transmissions do not overlap in time, from both the device’s and the cell’s viewpoint. The benefit of TDD is the channel reciprocity assumption, allowing improved channel estimation and link adaptation, including precoding and directional antennas. This is particularly beneficial for methods like beamforming.

**Figure 4:**
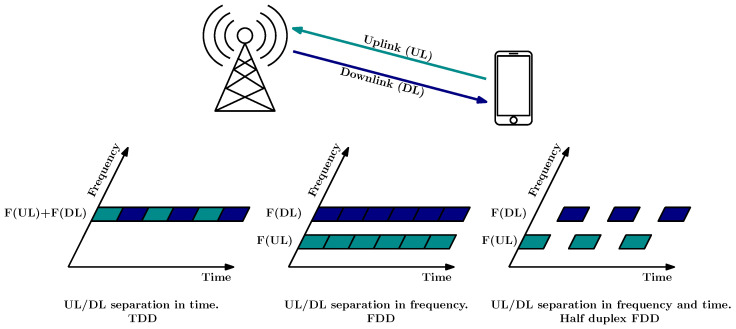
TDD mode vs. FDD in 5G NR.

Semi-Static TDD introduces a higher degree of flexibility compared to static TDD. In Time Division Long Term Evolution (TD-LTE), UL/DL configurations were defined within a single 10 ms frame. However, in 5G NR higher layer configuration parameters can be employed to achieve a UL/DL allocation parameterization that is specific to a cell or even a UE. Therefore, the slot configuration is adaptable and can be modified periodically while still prioritizing the management of inter-cell interference, see Figure 4.

Dynamic TDD is the most adaptable approach for configuring UL/DL. Signaling mechanisms play a crucial role in informing the UE about resource allocations for UL and DL transmissions. Firstly, dynamic signaling for the scheduled device involves the device monitoring DL control signals to determine whether to transmit in the UL direction or listen for DL transmissions, with the scheduler controlling the UL/DL allocation. Secondly, semi-static signaling via RRC allows for UL/DL allocation information to be transmitted, aiding in power conservation by reducing the need for constant device monitoring. Finally, dynamic slot-format indication shared among a group of devices utilizes a special downlink control message, the Slot Format Indicator (SFI), to dynamically signal UL/DL allocation, facilitating efficient resource management and channel quality assessments. These mechanisms collectively ensure adaptive resource allocation and effective traffic management in dynamic TDD scenarios. This method could be applicable for small cells, or even for standalone or isolated indoor cells that have overlapping coverage with neighboring cells, thereby reducing the impact of inter-cell interference.

#### 2.2.3. PHY Layer Channels

There is a list of PHY channels used in 5G NR. These channels play crucial roles in synchronization, system information, and overall communication.

- Physical Broadcast Channel (PBCH): This carries essential system information for UEs. It provides information such as downlink system bandwidth and timing information in radio frames, and it is part of Synchronization Signal (SS) burst set periodicity, system frame number, and other upper-layer details.
- Physical Downlink Control Channel (PDCCH): This carries essential control information called COntrol REsource SET (CORESET) that guides UEs on how to receive and decode downlink data. It provide information about information element multiplexing, channel coding, rate matching, scrambling, modulation, and dynamic resource control. Contrary to the 4G channel, it is not allocated across the entire system bandwidth and is more generalized.
- Physical Downlink Shared Channel (PDSCH): The main channel used for carrying user data from gNB to the UE. There are only minor changes between 4G and 5G.
- Physical Uplink Control Channel (PUCCH): This carries information such as Hybrid Automatic Repeat reQuest (HARQ) feedback, Channel State Information (CSI), and Scheduling Request (SR). In 5G, a short format to support low latency application is introduced.
- Physical Uplink Shared Channel (PUSCH): This carries data in the UL. It has the same function as in 4G.
- Physical Random Access Channel (PRACH): This is used by the UE to initiate random access procedures. The preamble contains information about the UE’s identity and timing adjustment. It allows the UE to request resources for UL transmission and establish synchronization with the network. Zadoff-Chu sequences are used to generate the random access preamble, similar to LTE technology.

With respect to the NR predecessor, the Physical Control Format Indicator Channel (PCFICH), carrying organization of data and control information in the downlink by Control Format Indicator (CFI), is removed. The Physical Hybrid ARQ Indicator Channel (PHICH), carrying hybrid Automatic Repeat reQuest (ARQ) indicators (ACKnowledged (ACK) Non-ACKnowledged (NACK)), is moved and is indicated in UL Downlink Control Information (DCI) in NR.

#### 2.2.4. PHY Layer Signals

The following signals in the physical layer of 5G NR are critical to enable effective communication between UE and the network:

- Primary Synchronization Signal: There are three possible sequences of the PSS. The Primary Synchronization Signal (PSS) is based on maximum length sequences (m-sequences), contrary to Zadoff-Chu sequences in LTE. There are 127 consecutive subcarriers in the frequency domain, contrary to 72 in LTE. The frequency position of the PSS in NR can vary in order to adopt more flexibility in the deployment, contrary to the fixed scenario in LTE. The PSS is linked to the cell identity group $N_{ID}^{2}$.
- Secondary Synchronization Signal: This is based on a Gold sequence of length 127 mapped to 127 subcarriers, which is formed by combining two m-sequences. Due to low cross correlation of signal, the UE can distinguish between neighboring base station. The number of possible Secondary Synchronization Signal (SSS) variations is 336, contrary to 168 in LTE. The SSS is linked to the cell identity group $N_{ID}^{1}$, and the signal length is the same as that of the PSS. Unlike LTE, the NR SSS does not change depending on which subframe it is transmitted from. Both PSS and SSS are related to the Physical Cell ID (PCI) by the following formula:
  (1) $$PCI=3\times N_{ID}^{1}+N_{ID}^{2}$$
  resulting in 1008 possible PCIs (also referred to as NCellIDs). This differs with respect to the LTE, where only 504 combinations are possible.
- DeModulation Reference Signals: These aid in the channel estimation for the coherent demodulation of PDSCH and PBCH, as outlined in [57]. DeModulation Reference Signals (DM-RS) symbols are mapped to specific resource elements within an RB. The structure depends on the network configuration, such as localized or distributed mapping and SCS. Contrary to LTE, the DM-RS are separated for the PDSCH, PUSCH, PDCCH, PUCCH, and PBCH.
- Phase Tracking Reference Signal: This is introduced in 5G PDSCH and PUSCH to help in the phase tracking process and to mitigate Common Phase Error (CPE) effects in the mmWave, caused by phase noise from local oscillators, ensuring system performance [57].
- Sounding Reference Signal (SRS): This is used for channel sounding in the UL. The signal is transmitted periodically by the UE and can utilize frequency hopping to avoid interference.
- Channel State Information Reference Signal (CSI-RS): This is transmitted by gNB to estimate DL radio channel quality. CSI-RS is used in beamforming to determine the best beam based on the channel’s state and to maximize the spectral efficiency in MIMO transmission [57].

### 2.3. Initial Access

#### 2.3.1. Basic Inital Access Procedure

**Figure 5:**
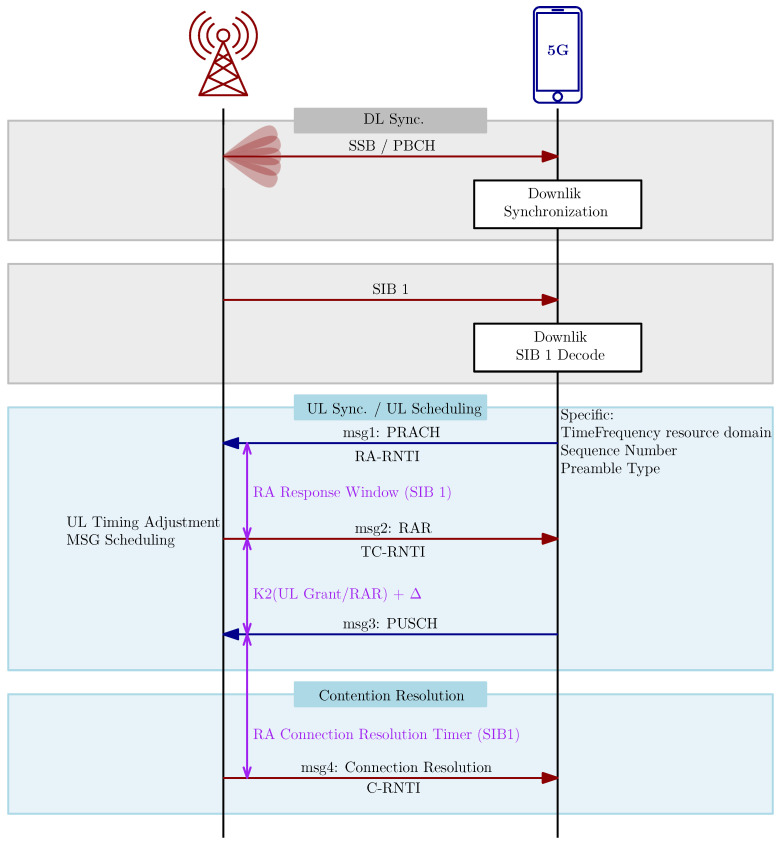
Initial access procedure and its messages. Parameters Δ and K2 specified in [60].

The initial access [59] of 5G is a sequential procedure between the UE and gNB to acquire UL synchronization and obtain identification information for the radio access communication; the process is also known as the Random Access Channel (RACH) process. This process is initiated in several cases. The initial access is called when the UE moves from the RRC idle state, which is related to the low-power mode, to the RRC connected state, or during the RRC connected state when the UL synchronization status is marked as non-synchronized, or during the RRC re-establishment procedure, or during the transition from the RRC inactive state. The procedure is also called when the UE demands system information that is not included in broadcast packets, or when the UE establishes a time alignment at the secondary cell to improve data rates or make a more reliable connection. The initial access procedure is also called in the case of beam failure or handover between cells. The initial access procedure is depicted in Figure 5. The primary distinction between LTE and 5GNR RACH occurs right before the RACH preamble transmission. This difference arises from the default support for beamforming in NR, particularly in mmWave scenarios.

#### 2.3.2. Beam Management in 5G

In NR, when operating in the beamforming mode, the UE must detect and choose the optimal beam for the RACH process. The Synchronization Signal Block (SSB) is closely related to beam management. SSBs are bursts of signals that contain essential information for the UE to perform initial cell search and synchronization with the gNB. In the context of beam management, SSBs are used to establish the initial beam-pair link between the UE and the gNB where the gNB transmits multiple SSBs, each on a different beam. The UE detects the best beam among them and sends PRACH to the location, which is mapped to a specific SSB beam ID. Beam management procedures are applied for both DL and UL transmission and reception [61,62]. These procedures include the following:

- Beam sweeping and Beam Alignment (BA) process: This involves broadcasting a series of beams across a defined spatial zone with predetermined timing and directional patterns. Each SS block corresponds to a specific beam.
- Beam measurement: This describes the assessment of signal reception quality at either the gNB or the UE, utilizing metrics like Reference Signal Received Power (RSRP), Reference Signal Received Quality (RSRQ), Signal to Interference and Noise Ratio (SINR), or Signal to Noise Ratio (SNR).
- Beam determination: This pertains to choosing the most appropriate beam or set of beams at the gNB or UE based on the data acquired from beam measurement activities.
- Beam reporting: This is a procedure during which the UE is communicating information about beam quality. The UE sends out the PRACH preamble that matches the SS block linked to the optimal beam. This direct correlation between the incoming SS block and the outgoing RACH preamble serves as the UE’s method of indicating the best beam choice to the gNB.
- Beam recovery: This is a process that detects beam failure and searches for another candidate beam with good quality. When the number of beam failures reaches the limit in RRC, the UE triggers the beam failure recovery process with the candidate beam using PRACH identified by a preamble index [63].

## 3. Possible Attack Types on Physical and MAC Layer

Since the 5G NR networks support various types of devices from low-power IoT devices, MTC, and smartphones working in a variety of frequency ranges and bandwidths, the NR technology has to support various types of protocol technologies and also inherit some technologies from their predecessor. Thus, 5G is susceptible to various types of attacks, such a Radio Frequency (RF) jamming, spoofing, and sniffing [19,56], with some of them being similar to the case of LTE. Several case studies on PHY layer security are presented in [64,65,66], along with several attacks and proposed countermeasures that have been realized in real-world scenarios, as published in [56,67]. An interesting study focused on higher-layer security and False Base Station (FBS), with examples of several attacks, and is available in [68]. The authors provide an example of a novel FBS attack including clock information injection and baseband fuzzing, which are manual and automated traffic injection methods that result in continuous DoS attacks.

The PHY vulnerabilities can be categorized into several groups, depending on which technology is used as the input point. Table 2 summarizes the selected attacks on the PHY and MAC layers. The table is sorted from less sophisticated attacks to more sophisticated ones. It is important to note that even smart jamming attacks need to be synchronized in most cases with the targeted gNB. The column “Effectiveness” represents the ratio between the attack’s efficiency and its complexity.

### 3.1. Jamming Attack Types

**Figure 6:**
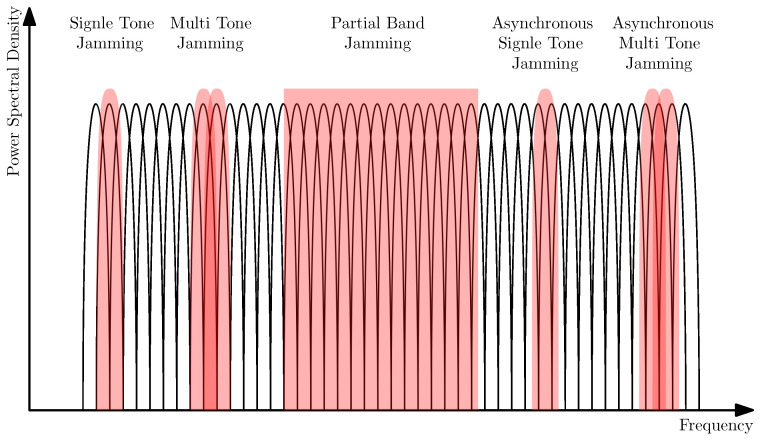
Illustration of jamming types with higher energy efficiency.

Traditionally, using band-limited noise to jam the entire transmitter band is considered as a functional but energy-inefficient jamming technique. More energy-efficient methods include partial band jamming, single-tone jamming, multi-tone jamming, asynchronous single-tone jamming, and asynchronous multi-tone jamming, as illustrated in Figure 6.

- Single tone jamming involves the utilization of a single, high-power Codeword (CW) tones to disrupt a single subcarrier. This method necessitates the precise knowledge of the subcarrier’s exact frequency, as depicted in Figure 6. Additionally, single-tone jamming can be employed to disrupt the cell-specific reference signal, consequently diminishing the overall system capacity. However, for effective jamming, the jammer must achieve perfect synchronization with the network.
- Multi-tone jamming is employed to disrupt multiple subcarriers simultaneously. In contrast to single-tone jamming, multi-tone jamming involves the generation of multiple random phase CW tones [69]. Similar to single-tone jamming, multi-tone jamming also necessitates accurate knowledge of the subcarriers’ exact frequencies, as illustrated in Figure 6.
- Partial band jamming involves transmitting Additive White Gaussian Noise (AWGN) across a specific frequency band. The effectiveness of this jamming technique correlates directly with the ratio of the jamming bandwidth to the signal bandwidth when maintaining constant jamming power [70,71]. As depicted in Figure 6, this method allows for the jamming of a segment of continuous subcarriers.
- The asynchronous form of jamming can be categorized into two types: asynchronous single tone jamming and asynchronous multi tone jamming, as also shown in Figure 6. The underlying principle of asynchronous jamming involves disrupting the target signal with a frequency offset from the subcarrier. This offset allows the jamming signal to interfere with neighboring subcarriers, resulting in a scenario of Inter-Carrier Interference (ICI) at the receiver, rather than directly jamming the signal. Compared to the other types of jamming mentioned earlier, asynchronous jamming demonstrates superior performance [72].

The susceptibility of a physical channel or signal to jamming depends significantly on its sparsity within the overall time–frequency resource grid [19]. A key factor mitigating vulnerability is if the channel or signal is allocated on the time–frequency resource grid using a dynamic scheme controlled by higher-layer parameters, which may not be known to a potential jammer. This fact, from the point of view of the jammer, defines a trade-off between the complexity of the jamming method and the effectiveness of the jamming and jammer power consumption. Also, Frequency Ranges (FR) are a factor that have to be calculated in jammer design, since the hardware complexity of FR2/mmWave jammers is significantly greater than the jamming of the sub 6 GHz channels.

### 3.2. Smart Jamming

Knowing the frame structure of 5G transmissions, smart jamming attacks can be targeted against various physical signals in both UL and DL directions, as described bellow.

- Synchronization signals in NR are more resilient to jamming [56] due to Gold sequence low cross correlation allowing the UE to distinguish between nearby base stations on the same channel at low SINR. Thus, jammers need to transmit with high power in order to jam the synchronization signals. The SS bursts are mapped to the resource grid based on SCS, carrier frequency, and offset-ref-low-scs-ref-PRB parameters [57]. A jammer targeting the PSS and/or SSS in time needs to synchronize with the cell and identify the SCS, which can often be determined using public band plans. This is slightly more complex than in LTE [19].
- The Physical Broadcast Channel (PBCH) is located in the same slot as PSS and SSS. The combination of PSS/SSS with PBCH, known as SSB, is transmitted over 4 OFDM symbols and 240 subcarriers (20 RBs). The Synchronization Signal Block is transmitted with a variable interval with at least a period of 20ms, depending on SCS. The allocation in time–frequency space is variable to the center or to the side of the DL channel. Jamming the PBCH prevents the UE from accessing the critical information it needs to connect to a cell. If the jammer can synchronize with the target cell, PBCH jamming can be done in a time-selective manner. Alternatively, the jammer could continuously jam the subcarriers on which the PBCH is located. The latter method involves jamming 240 subcarriers. To put this into context, a 20 MHz DL with a 15 kHz SCS has 1272 subcarriers. Therefore, this would mean jamming approximately 19% of the DL signal, resulting in a jamming gain of approximately 7 dB compared to barrage jamming [56,73].
- The Physical Downlink Control Channel (PDCCH) is used to send control information to the UE to schedule DL and UL transmission and defines its modulation and coding and carries HARQ and DCI messages. The PDCCH always starts in the first symbol of each slot, is Quadriphase Phase Shift Keying (QPSK) modulated, and uses polar coding. To determine PDCCH localization in the time–frequency grid, the CORESET parameters have to be demodulated [57]. To successfully jam the PDCCH, the jammer has to set a valid jamming duty cycle according to the CORESET time duration parameters [56]. DCI is vulnerable to various passive attacks, such as localization and traffic fingerprinting, which infer users’ information from allocated resources.
- The Physical Uplink Control Channel (PUCCH) carries control information about the Scheduling Request (SR), HARQ, UL CSI, and others [74]. There are five formats of PUCCH carrying various parameters provided by higher layers. The PUCCH is modulated by Binary Phase Shift Keying (BPSK) or QPSK and can be coded by polar or simplex or Reed Muller codes. PUCCH can also implement inter slot hopping to reduce interference. Similarly to LTE, some control information can be carried by PUSCH. This makes PUCCH very unreliable for jamming.
- Reference signals are used for the channel estimation of DM-RS or for phase tracking the Phase Tracking Reference Signal (PT-RS). DM-RS can be assigned in both the time and frequency domains, and they are separated by physical channels; compared to LTE, DM-RS are assigned in the frequency domain and are transmitted in specific resource blocks within the LTE carrier. For the jammer, the ideal Reference Signal (RS) to disrupt is the one that requires minimal energy, but that is crucial for the link’s operation. The DM-RS for the PBCH fulfill the criteria as it is consistently located and only needs the cell ID and PBCH location, which a time-synchronized jammer can easily know. The DM-RS for the PBCH takes up a quarter of the PBCH’s Resource Elements (REs) and can be jammed without cell synchronization by disrupting the correct 60 subcarriers. PT-RS for the PDSCH are used only when the higher layer parameter is enabled. The mapping depends on time density and frequency density parameters. The effectiveness of a downlink PT-RS jamming attack is uncertain without knowing how often PT-RS are enabled and the density default set by base station vendors [56].
- The Downlink and Uplink User Data Physical Downlink Shared Channel (PDSCH) and the Physical Uplink Shared Channel (PUSCH) represent the main part of the frame. It is feasible to selectively interfere with these channels. The jammer could equally interfere with the whole UL and DL; hence, the jamming of PDSCH and PUSCH is one of the least important threats to consider.
- The Physical Random Access Channel (PRACH), similarly to LTE, is used by the UE after the synchronization of SSB and the decoding of PBCH to transmit a preamble that is in the form of a Zadoff-Chu sequence that embeds a value used for the temporary identification of the UE. The gNB broadcasts the candidate locations of the PRACH in the time–frequency grid in case the UE attempt to connect [57]. From the jammer point of view, the many configurations of PRACH and real-time data decoding makes the jamming of PRACH unreliable.
- Another published attack exploits the beam configuration of a cell to proceed a SSB-RA fingerprinting localization attack. The attacker creates a map detailing the exact locations of the BS and all beams within a cell. By monitoring the random access channel, the attacker can infer the beam selected by the UE from the random access occasion. The attacker also acquires the Timing Advance (TA) value from the RA response sent by the BS. These values are then used to calculate the UE’s azimuth and distance from the BS, providing an estimated location of the UE. With a combination of PDCCH Order (PO), the attacker can also target already connected users.

To quantify the complexity and power efficiency of jamming-type attacks against the 5G PHY layer, the Jammer to Signal (J/S) ratio was introduced in [19,75]. According to Table 3, considering the complexity of attacks on PHY channels, the most effective jamming or spoofing attacks are those in DL against PSS, PBCH, SSS, and DM-RS of PBCH, respectively. The less effective attacks in DL are against PDCCH, and in the UL against PRACH and PUCCH.

### 3.3. Spoofing

**Figure 7:**
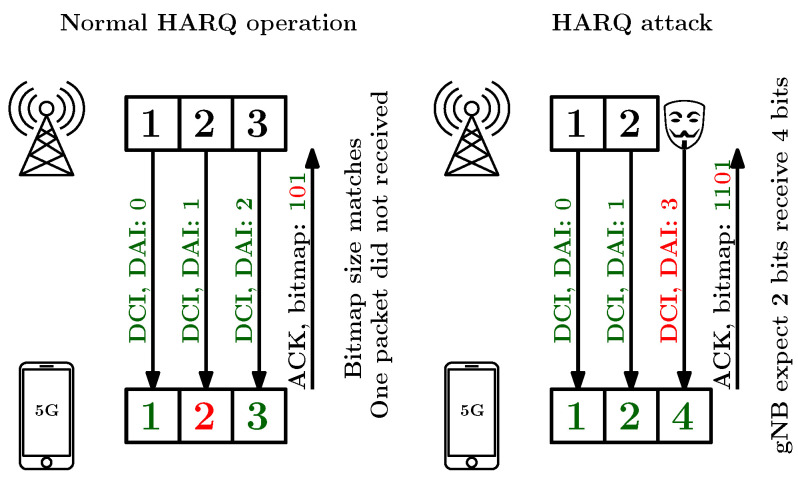
Hybrid Automatic Repeat reQuest attack in PDCCH.

- Synchronization signal spoofing is a more effective attack in contrast to a simple injection of wideband noise by the jammer. Attackers are able to effective transmit fake PSS/SSS signals, as it does not require cell synchronization and requires less power. Note that PSS and SSS are detectable at low SNR [19], and their successful jamming would require a higher J/S ratio. Spoofing involves transmitting asynchronous fake PSS signals at higher power, potentially causing denial of service during the initial cell search [76]. The 5G NR specifications do not detail UE behavior upon detecting a valid PSS without an associated SSS [75], making the impact of PSS spoofing implementation specific. More sophisticated blacklisting is needed to mitigate the effects of increased fake PSS transmissions.
- Physical Broadcast Channel (PBCH) sniffing and spoofing can be processed similarly to the LTE [19]. A Master Information Block (MIB), providing information about System Information Block (SIB) mappings in the time–frequency grid, represents the information that can be sniffed and spoofed. SIB provides information such as the idle timer configuration of the network, unique identifiers of the cell, and the RB mapping of critical control channels, and it also provides information on the received power threshold that can trigger a handover to another cell. Contrary to LTE, the NR SIB and RRC messages introduce new parameters, such as a whitelist or blacklist of cells. These unprotected messages can be exploited for security breaches against the NR protocol by spoofing SIB messages or impersonating a base station during the RRC handshake [67,77].
- Physical Downlink Control Channel (PDCCH) spoofing is analyzed in [67], which describes several vulnerabilities linked with DCI carried by PDCCH, such as the following:
  - Attacks on resource scheduling: For the DL, this has limited value as the UE fails to decode data in incorrect slots. However, for the UL, it is more effective as an attacker can cause multiple UEs to transmit over the same resources, leading to jamming and battery drain. The attacker crafts and injects UL DCI into each time slot, making Induced-Jammer UEs (IJ-UEs) transmit data, even without pending data. To amplify the attack, the attacker manipulates the Transmit Power-Control (TPC) field within the same DCI to force the IJ-UEs to transmit at maximum power. This severely impacts the SNR of other devices and reduces their throughput.
  - PDCCH Order (PO): This is a special DCI that instructs a specific UE to initiate an RA procedure to update synchronization. As the only unprotected control procedure for triggering RA, it efficiently and stealthily induces RA, causing resource drain and potential disconnections and can be used for triggering localization attacks.
  - Bandwidth Part (BWP) Switching Attack: This exploits a 5G feature dividing total bandwidth into multiple parts for different users. An attacker can spoof this DCI, redirecting the UE to different BWP, causing loss of scheduled transmissions and disrupting connectivity. This can also facilitate sophisticated attacks like Man-In-The-Middle (MITM), where specific messages from the base station are missed, or DL data are injected into an empty BWP.
- Physical Uplink Control Channel (PUCCH) spoofing vulnerabilities have been published in [67], where the authors provide several attacks against PUCCH, such as the following:
  - Spoofing Scheduling Request (SR) exploits the UL physical layer message sent from the UE to the gNB to request UL resources. When the gNB receives an SR, it allocates resources for the user. SRs can be exploited by attackers in three ways: An attacker can keep users’ Radio Network Temporary Identifier (RNTI) connections active for extended periods, bypassing the RRC inactivity timer and enabling long-term tracking. They can request resources on behalf of multiple users without pending UL data, leading to network congestion. Furthermore, they can request an UL DCI for a specific user and hijack the allocated UL grant to spoof higher-layer data on behalf of the user. The second method is a stealthier alternative to the resource scheduling attacks mentioned earlier since spoofed UL transmissions are harder to detect as they may appear to come from legitimate UEs. The third method is particularly advantageous for attackers as it allows them to hijack UL grants and inject MAC layer information for a specific user on demand.
  - The HARQ attack exploits the lack of synchronization between the base station and a UE due to spoofed DCI with an altered Downlink Assignment Index (DAI). An attacker injects a DCI with a higher DAI value than expected, causing the UE to report an incorrect ACK bitmap size. This mismatch leads to a HARQ failure, as the base station cannot match the ACKs to the correct packets, resulting in communication disruption and potential loss of connectivity for the targeted UE. This attack is effective because it leverages the unprotected nature of the lower protocol layers, where integrity checks are not enforced. This attack is depicted in Figure 7.
  - Channel State Information (CSI) sniffing to track users. This attack uses the vulnerability of the L1 layer combined with the L2 layer and allows tracing of users by SRS or leakages in CSI-RS [67]; more information is described in Section 4.
- Contrary to jamming, the spoofing of Physical Random Access Channel (PRACH) signals can be applied in flood attacks, where the transmitting of large numbers of invalid preambles can be used for DoS attacks on gNB. The authors in [67] applied an attack against initial access using the SIB overshadowing technique by modifying the *ra-ResponseWindowSize* parameter to minimum and the *preambleTransMax* parameter to maximum, resulting in a failure for all UEs trying to connect to the network. To amplify the collision effect during RA, the PO can be injected to multiple users. This type of attack also effects UE battery draining because the UE increases transmit power after each unsuccessful RA.

### 3.4. Jamming and Spoofing Mitigation Techniques

Several techniques have been proposed to mitigate the effects of spoofing attacks to the 4G/5G PHY layer, such as:

- PSS spoofing can be mitigated using a timer and a blacklist. After a certain time when the SSS is not received, the PSS is blacklisted for a specific time and the second strongest PSS at the channel can be chosen.
- PSS/SSS spoofing can be mitigated by the UE’s proactive measures. The UE can generate a comprehensive list of all the cells present within a specific frequency channel. This list should also include the received power levels of each cell, as described in [78]. Then, PBCH can be decoded for the strongest cell and timer applied for decoding MIB. After timer expiration, the second strongest cell can be decoded.
- Spoofing and sniffing can be mitigated by the reduction of information broadcast by MIB and SIB frames, which contain essential information to establish a radio link [79]. Both UEs and base stations implicitly trust all messages before authentication and encryption, potentially leading to security exploits. It is essential to develop methods for UEs to verify a base station’s legitimacy before acting on unauthenticated RRC and NAS messages, despite current specifications not requiring this.

## 4. mmWave and MIMO-Specific Attacks

### 4.1. Beam Alignment Jamming

**Figure 8:**
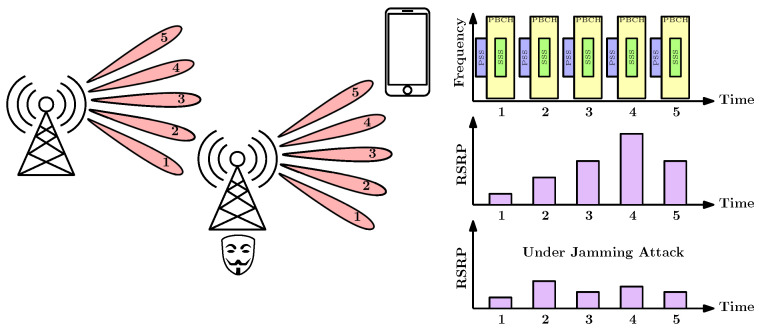
NR beam-alignment procedure and jamming attack.

The BA process outlined for 5G NR has been engineered to be fast and precise in non-malicious radio environments [62]. However, it can represent a potential vulnerability and advantage for smart jammers [80,81]. They could initiate an attack during the BA phase with the aim of reducing the precision of beam selection. This could negatively affect the overall performance and the quality of service that users experience. This type of attack can also be performed on other communication standards relying on beamforming technology, such as 802.11ad [82]. The 5G NR BA process is illustrated in Figure 8.

In order to mitigate the effect of the BA jamming attack, the authors of [80] propose a randomized probing technique, which involves transmitting a corrupted probing sequence to enable the user equipment to reject the jamming signal using subspace-based orthogonal projections and jamming cancellation. This method shows promising results in maintaining the accuracy of beam selection and ensuring the quality of service despite the presence of smart jammers.

### 4.2. Pilot Contamination

Another type of vulnerability could be pilot contamination. The term pilot contamination refers to the interference that occurs in the channel estimation process when the same pilot sequences are reused in adjacent cells or by injection by the attacker. This is a significant challenge in massive-MIMO (mMIMO) systems because the size of the coherence interval is constrained, limiting the length of the pilot sequence. In [83], the authors modify the precoder employed by the legitimate transmitter in a controlled way to strengthen signal reception at the eavesdropper during data transmission. Additionally, they explore the transmission efficiency of an advanced full-duplex eavesdropper to ensure effective eavesdropping and impair the detection capabilities of the legitimate receiver simultaneously.

### 4.3. User Tracking

In [67], the authors identified several vulnerabilities in L2 MAC procedures with a combination of NR MIMO technology. One of the vulnerability is the potential to track users using SRS reference signals. SRS are applied to report channel state information, and for resource allocation optimization. SRS can be transmitted periodically, semi-persistently, or a-periodically. These modes are driven by secured RRC messages. Semi-persistent reference signals are sent at regular intervals and can be enabled or disabled via an MAC Control Element (Semi-Presistent (SP)-SRS Activation/Deactivation) [67]. Aperiodic reference signals, on the other hand, are initiated by DCI and require a single transmission. Here is an overview of the most important related threats:

- Tracking using SRS signals: SRS is applied for assessing the channel conditions over the entire UL frequency range and for transmitting UL mMIMO pilot signals. The attacker can use this in three ways. Firstly, since it is an unexpected UL transmission by the BS, it can cause interference with other UL communications arranged by the BS. This interference results in either the jamming of user data or contamination of other users’ SRSs, leading to a disruption in the CSI that has been gathered. Secondly, an attacker can interrupt ongoing semi-persistent SRS transmissions by issuing a deactivation MAC Control Element to a UE. This action can significantly impair the channel estimation process at the BS, which in turn can drastically reduce the data throughput for the UE, especially in MIMO scenarios where beamforming is utilized. Lastly, the SRS is composed of a pre-established wideband Zadoff-Chu sequence, known for its excellent cross-correlation characteristics. Despite its original design not being intended for localization purposes, an attacker could exploit this signal to pinpoint the location of a specific user with a high degree of precision by measuring the differences in signal arrival times [84].
- Leakages in CSI-RS: DL channel measurement differs from SRS. CSI-RS signals are sent by the base station, and the UE reports them back. This report includes beam identifiers and signal-strength RSRP, which can be used by an attacker to track user locations. The attacker first fingerprints the cell beam layout, then decodes the UE’s CSI report to obtain beam information. The authors in [67] applied a *BeamToPath* algorithm to outlier sporadic signals and discarded impossible beam transitions. Finally, they computed path coordinates and interpolated the path for location tracking with 14 m in 90.32%, with an average error of 5.34 m, compared to the Global Positioning System (GPS)-recorded path.
- Beam Failure Recovery (BFR) DoS: Beam management requires rapid, dynamic reconfiguration to respond the changes in the wireless environment, like signal blockage. Typically, the gNB triggers beam management processes, such as beam swaping. However, if a UE detects beam failure, it measures the strongest beam and initiates an RA procedure, including the BFR MAC control element in Msg3, signaling that the user is switching to a different beam. This process can be exploited by an attacker, causing a misalignment between the beams at the UE and gNB, since the legitimate UE is unaware of the beam switch.

With the standardization efforts towards the 5G-Advanced and Sixth Generation (6G) of mobile communications, the Reconfigurable Intelligent Surfaces (RIS) PHY layer security must not be neglected in order to ensure secure and reliable communications in advanced wireless networks. Metasurface manipulation attacks pose potential vulnerabilities to RIS in terms of jamming [85], eavesdropping [86], pilot spoofing [87], and pilot contamination [88] attacks.

### 4.4. MIMO and mmWave Attacks Mitigation Techniques

In order to mitigate or detect jamming and spoofing attacks in NR FR2, several methods have been published. These methods typically use virtual channel [89] representation, which effectively describes the spatial properties of the channel.

In [80,90], the authors proposed a randomized probing technique, which involves transmitting a corrupted probing sequence to enable the user equipment to reject the jamming signal using subspace-based orthogonal projections and jamming cancellation. This method shows promising results in maintaining the accuracy of beam selection and ensuring the quality of service despite the presence of smart jammers.

In [91], the authors applied a Principal Components of Channel Virtual Representation (PC-CVR)-based method to detect spoofing attacks. The method uses a statistical test for static environments and machine learning for dynamic environments—achieving high accuracy in both scenarios. The non-machine-learning is introduced in [92], where the authors applied a more simplified method based on leveraging the sparseness and statistical features of virtual channels.

In [93], the authors proposed an active–passive cascaded RIS-aided receiver for jamming nulling and signal enhancing based on a low-complexity optimization framework using Alternating Majorization-Minimization (AMM) and Conventional/Modified Cyclic Coordinate Descent (CCD) (C/M-CCD) methods to obtain the coefficients of the active RIS. Another method is proposed in [94], where the algorithm forks in two phases. Firstly, uncertain jamming information is robustly processed using CCD and Successive Convex Approximation (SCA) to optimize the RISs’ phase shift and amplitude matrices. Additionally, closed-form solutions for the transmitting and receiving beams are derived. Finally, a low-complexity Block Coordinate Descent (BCD) algorithm alternately optimizes these variables and a greedy algorithm manages and adjusts the multiple RISs.

## 5. Test-Beds for 4G/5G Experimentation

To verify vulnerabilities at the PHY layer, a proper testing system is necessary. The development of a full 4G/5G systems is a very time- and money-consuming process; fortunately, there are currently possibilities to build a private 4G/5G network for reasonable costs. For such implementations, three components are needed: the Software Defined Radio (SDR) with well-chosen front-end, which serves as the transmitting and receiving device; the RAN, which manages the wireless communication between mobile devices and the backbone network; and the backbone network, which includes all 5G functions and interactions including authentication, security, session management, and traffic aggregation from end-user devices. Various Software (SW) frameworks and tools have been developed for experimenting with security threats and countermeasures in 4G/5G cellular networks [95]. The open-source projects developed by the community are one of the most suitable solutions for such experiments. Here, the functionality of the overall system can be adapted to demonstrate vulnerabilities or implement countermeasures against specified threats. There are several options for RAN implementation, including, but not limited to the following:

- Free5G RAN [96] (Hsinchuis, Taiwan, current version v3.4.2) an open-source project with very limited functionality. The current version includes a receiver that decodes MIB and SIB1 data and can act as a cell scanner in SA mode.
- OpenAirInterface [97] (Biot, France, current version v2.1.0) developed by Eurecom, and maintained under a public license, which implements NSA/SA gNB as well as 5G NSA/SA UE. The project is also integrated with the NVIDIA Aerial software development kit’s L1 inline hardware accelerator with the OpenAirInterface L2 and above to build an accelerated 5G virtual RAN. This kit provides a full L1 high-PHY implementation of 5G NR compatible and interoperable with the Open-RAN 7.2× front-haul split.
- srsRAN [98] (Cork, Ireland, current version 23.11) an open-source SW that provides a compete 5G RAN solution, optimized for SDRs [99]. This software package supports 5G NSA/SA modes for both srsUE and srsENB. In 4G mode, it implements the EPC; alternatively, for 5G SA mode, it supports the 3rd party 5G cores. The big advantage of srsRAN for amateur and educational usage is that it can be used with the ZMQ virtual RF front-end. In such a case, there is no need for physical RF-hardware to implement RAN. It allows simultaneous packet capturing and analyzing by a Wireshark. There have been many recent research publication profiting from the use of this project [19,67,100].

According to provided tests [99], srsRAN is more suitable for beginners in the field but yields unstable results in terms of latency, while OpenAirInterface offers more flexible configuration options. A brief overview of related works dealing with SW tools developed to test the vulnerabilities of cellular protocols is provided in [101].

These findings highlight the importance of selecting the appropriate software tools for testing and implementing 5G technologies. There are many options for 5GC implementation. Some include only 5GC for 5G SA networks, while others also include EPC for 4G and 5G NSA modes. The most known and widespread are as follows:

- Open5GS, an open-source project that provides a comprehensive 4G/5G core network solution. It implements a Release 17-compliant EPC for 4G and 5G NSA networks, as well as a 5G SA core. Open5GS supports the delivery of voice calls and text messages through the LTE network. This is achieved by leveraging third-party Voice over LTE (VoLTE) and SG-SMS (SMSoSGs) solutions, respectively, such as those from Kamailio and Osmocom [102]. This 5GC is recommended by srsRAN for 5G SA.
- free5GC, an open-source 5G mobile core network project. The goal of the project is to implement the 5G core network defined in 3GPP Release 15 and beyond [103].
- Open5GCore toolkit, the first global practical implementation of the 3GPP 5G core network, supporting the functionalities of 3GPP Releases 17 and 18. Open5GCore implements the new 5G components as a standalone, independent of the previous 4G EPC functionality [104].
- Open Core Network is a cloud-native and converged core that consists of a collection of microservices implementing various core network functions. Supports 3GPP 5GC and LTE EPC for licensed, unlicensed (e.g., Wi-Fi), and shared spectrum (e.g., CBRS) networks. It enables seamless migration from 4G EPC to 5GC in both NSA and SA modes [105].

Based on chosen RAN implementation and the selected mode of operation (4G or 5G NSA/SA), a suitable SDR has to be chosen to be used for UE, gNB/Evolved Node B (eNB) implementation. In the case of OpenAirInterface, these devices are supported: Ettus USRP B2x0/X3x0 families, or a proprietary solutions EURECOM ExpressMIMO2 [106], and the capabilities of individual SDR’s are shown in Table 4. In the case of srsRAN, these devices are reported as supported: Ettus USRP B2x0/X3x0 families, BladeRF, and LimeSDR [98]. For details on the capabilities of individual SDR’s, see Table 5.

Our team experimented with srsRAN [107,108], where the raw data are captured by a modified RFSoC development kit from AMD/Xilinx [109] with an XM500 frontend.

## 6. Machine Learning for Enhancing PHY Layer Security

Advances in machine-learning methods, both classical [110] and especially deep learning, have opened up opportunities for use in enhancing the security of 5G networks. To secure the wireless transmitters at the physical layer, exploitation of machine-learning techniques mainly leverages either the unique RF impairments of transmitting devices (RF fingerprinting) or channel state information inconsistencies via anomaly detection or model-based approaches. In the following, we provide an overview of the most-used machine-learning and deep-learning models and outline their use for physical layer-based authentication.

### 6.1. Convolutional Neural Networks

Convolutional Neural Network (CNN) are one of the most fundamental architectures in the field of deep learning [111]. Initially inspired by the human visual perception mechanism [112], they have been proven to be highly effective for computer vision [113]. Later, they were adopted for several other tasks, such as natural-language processing [114], automatic modulation classification, signal identification, and interference detection and RF fingerprinting for LTE and 5G networks [107,115,116].

**Figure 9:**
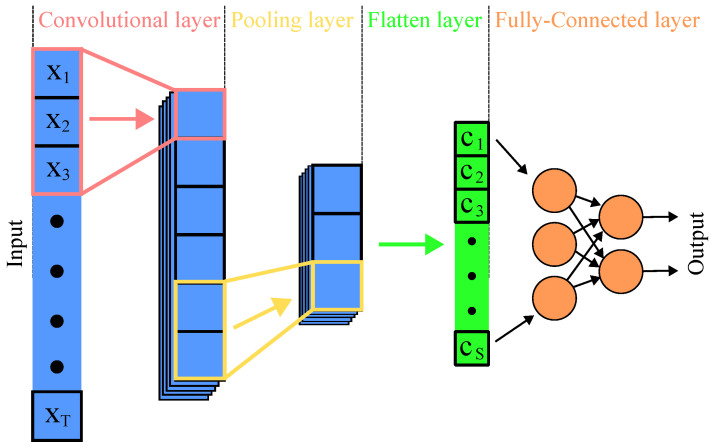
Schematic of a simple CNN for processing 1D input data with depicted *Convolutional*, *Pooling*, *Flatten*, and *Fully-Connected* layers. The input data are vectors *X* with length *T*. Figure from [120] and extended.

The CNN typically consists of several layers that process and transform the inputs to produce the desired output, where the fundamental layers are called *Convolutional layers*, *Pooling layers*, *Flatten layers*, and *Fully-connected layers* [117]. Convolutional layers are the heart of CNNs and consist of learnable convolutional kernels that extract and learn features automatically through convolution operation. Pooling layers are used to reduce the spatial dimensions of the input data and help reduce the computational load and memory usage. On top of that, they help to detect invariant features to scale and orientation changes. Towards the end of the network, CNNs typically include one or more fully connected layers (equivalent to traditional Multi-Layer Perceptron (MLP) networks), which perform high-level reasoning, such as classifying the input data based on the features extracted by the Convolutional and Pooling layers [111]. The schematic of a simple CNN for the processing of 1D input data is shown in Figure 9. Table 6 presents recent examples of CNN-based machine-learning experiments applied to 5G devices security enhancements in terms of device authentication by fingerprinting, or jamming attack detection. The potential of CNN architecture has also been studied for satellite transmitters [118] and represent a pioneering work towards the use of future 5G non-terrestrial networks [119].

### 6.2. Triplet Networks

**Figure 10:**
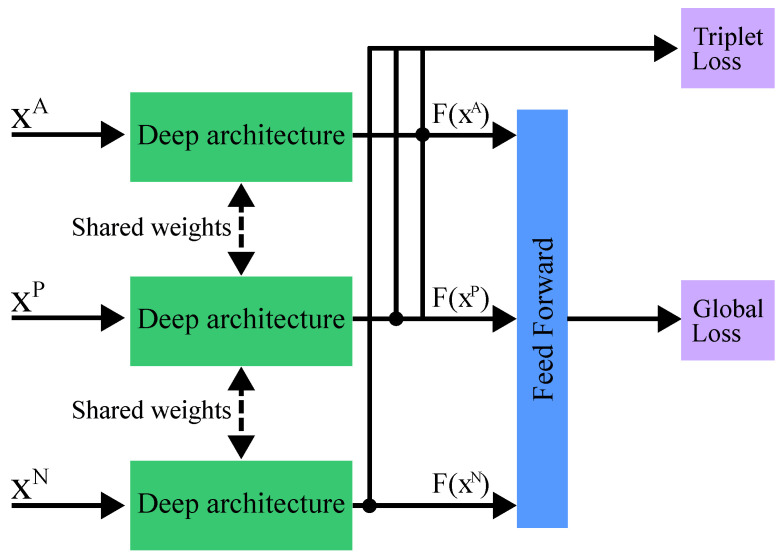
The general structure of the triplet network. The network has three inputs—an anchor signal $\left(x^{A}\right)$, a positive signal $\left(x^{P}\right)$ and a negative signal $\left(x^{N}\right)$. The inputs are passed through concurrently in parallel through the same structures Deep architectures *F* with shared weights resulting in embeddings $F(x^{A})$, $F(x^{P})$ and $F(x^{N})$. The embeddings are directly used to calculate the triplet loss, and extra Feed Forward layers are used to calculate the global loss. The final loss is an addition of triplet loss and global loss.

A triplet network is a type of neural network architecture designed to learn the similarity of fine-grained input data in an embedding space. The general network architecture is shown in Figure 10. The core idea behind a triplet network for RF fingerprinting is to take three input signals at a time, referred to as a triplet: an anchor signal $\left(x^{A}\right)$, a positive signal $\left(x^{P}\right)$—similar to the anchor, and a negative signal $\left(x^{N}\right)$—dissimilar to the anchor. These signals are passed through a shared neural network, which generates embedding (vectors) for each image.

The objective of the network is to learn embeddings so that the distance (often Euclidean or cosine similarity) between the anchor and the positive example is smaller than the distance between the anchor and the negative model by a margin. This is achieved through a loss function known as the triplet loss. The triplet loss is formulated to ensure that, for a given anchor, a positive example of the same class is closer to the anchor than any negative example of a different class. The triplet loss is typically combined with the global loss function, which can be a standard classification loss function, such as categorical cross-entropy. The network can be built on any deep-learning architecture, *F*, such as CNN, that typically share weights across the parallel architecture branches, as depicted in Figure 10 [125,126].

The triplet function built upon CNN has been successfully implemented and demonstrated to achieve an accuracy of 99.86%, irrespective of the training/testing time gap for the over-the-air datasets [126].

### 6.3. Reccurent Neural Networks and Long Short-Term Memory

Recurrent Neural Network (RNN) and Long Short-Term Memory (LSTM) [127] networks represent advancements in deep learning for analyzing and interpreting sequential and time-series data, potentially in applying RF fingerprinting.

**Figure 11:**
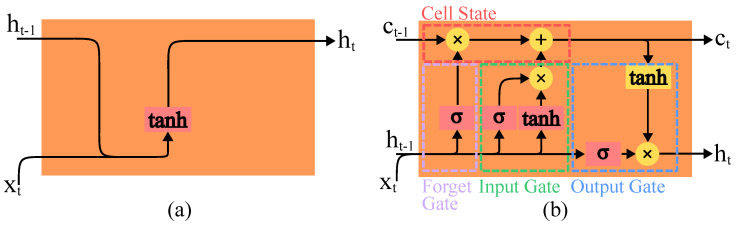
The fundamental building cells of (**a**) RNN and (**b**) LSTM networks. $c_{t}$ denotes the cell state, $h_{t}$ is the current hidden state, and $x_{t}$ is the input data. $h_{t-1}$ and $c_{t-1}$ are the previous hidden and cell states, respectively. The yellow blocks are component-wise and the red blocks are layers. LSTM has marked the *Forget Gate*, *Input Gate*, and *Output Gate*.

RNNs are designed to process data sequences by maintaining a hidden state that acts as memory and allows them to capture temporal dependencies and patterns within the data. However, RNNs often struggle with long-term dependencies due to issues like vanishing and exploding gradients. LSTMs, a special type of RNN, address these challenges with a more complex internal structure comprising gates that regulate the flow of information. These gates effectively allow LSTMs to remember and forget information over long sequences, making them particularly adept at modelling time-series data or sequences where the timing and order of events are critical [128,129]. The fundamental building units of RNN and LSTM networks are depicted in Figure 11.

The capabilities addressed above make RNNs and LSTMs particularly suitable for RF fingerprinting applications that require analysis of the temporal characteristics of signals, enabling more accurate identification of devices and transmission patterns within LTE and 5G networks. Integrating RNN and LSTM models in RF fingerprinting signifies a move towards more sophisticated, temporal-sensitive analysis techniques, offering improved performance over traditional methods in capturing the dynamic nature of wireless signal transmissions. To our best knowledge, although an LSTM has very recently been used for 5G anomaly detection in signaling traffic [130], no works have used RNNs and LSTMs for the RF fingerprinting of 5G and LTE signals on the PHY layer. Nevertheless, several recent works have focused on other wireless transmission signals. For instance, [131] focuses on fingerprint identification for Long Range (LoRa) devices and [132] considers a set of USRP SDR’s transmitting WiFi signals. The 802.11a/g signals transmitted from USRP have also been considered in [133], with the neural network architecture consisting of convolutional-LSTM architecture. At the same time, multiple deep-learning approaches, MLP, CNN, and LSTM, are compared with multiple variants of the input data, such as raw In-Phase Quadrature (IQ), frequency domain signal, and spectrogram. Study [134] is based on processing raw IQ data for RF fingerprinting by software-defined radio-type USRP-2900.

Besides direct use for the authentication of devices, recursive networks also have the potential to improve the performance of location-based approaches, such as the RNN used for signal preprocessing in [135].

Although not yet showcased for 5G, the properties of the RNN and LSTM networks described above, and successful demonstrations of RF fingerprinting presented in papers [131,133,134], make these network architectures potential candidates for successful RF fingerprinting classification of LTE and 5G transmitter devices.

#### Transformers

**Figure 12:**
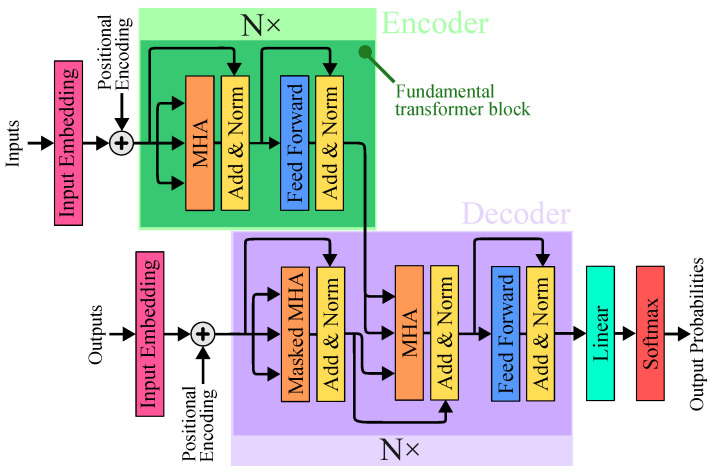
The vanilla Transformer architecture from [136]. The fundamental building blocks are the *Input Embeddings*, *Positional Encoding*, *Encoder* (green block), and *Decoder* (purple block). The orange blocks represent the Multi-head self attention (MSA) modules, the yellow blocks are the additions of residual connections and normalization layers, and the blue blocks are the feed forward neural networks.

Transformers, initially introduced in [136] in the context of Natural Language Processing (NLP), have rapidly become a revolutionary architecture in the field of deep learning due to their ability to handle sequential data without the limitations inherent to RNNs and LSTMs networks [137]. The schema of vanilla Transformers is depicted in Figure 12. At the core of the Transformer architecture is the self-attention mechanism, commonly extended to multi-head self-attention, which allows the model to weigh the importance of different parts of the input data, enabling it to process sequences in parallel and capture complex, long-range dependencies more effectively than its predecessors [138]. This characteristic is particularly beneficial for RF fingerprinting, where capturing the intricate temporal and spatial relationships within signal data can enhance identification accuracy and robustness in LTE and 5G networks. The ability of Transformers to efficiently handle large data sequences while maintaining context awareness makes them a promising option for RF fingerprinting methodologies. As far as we are aware, there has yet to be a recent publication on research focusing on RF fingerprinting utilizing 5G/LTE signals. However, a few recent studies have explored similar concepts using alternative technologies. The approach from [139] used LoRa devices, where the input of the model consisted of spectrograms obtained via *Short-Time Fourier transform (STFT)*. The collected signals were preprocessed, and the preprocessing compromised four stages: (1) Synchronisation, (2) Preamble Extraction, (3) Carrier Frequency Offset (CFO) Compensation, and (4) Normalization.

### 6.4. Autoencoders

**Figure 13:**
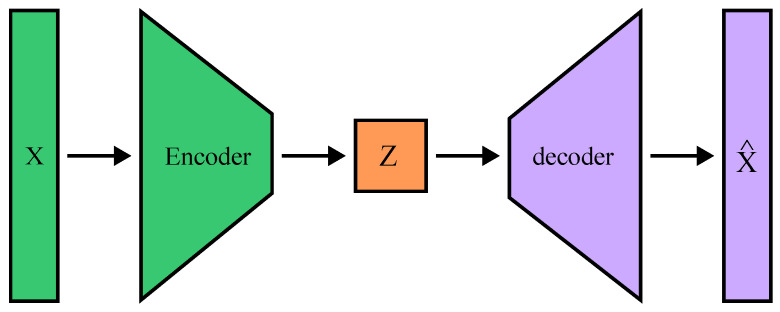
General autoencoder architecture.

From the point of view of deep-learning, tamper detection in LTE and 5G networks can be treated as an anomaly detection task. An anomaly detection task identifies unusual patterns or outliers in data that deviate from the norm. To our knowledge, only a limited number of recent papers have been published, so this section will review potential deep-learning architectures for anomaly detection and, if applicable, survey the recent works. The main goal of an autoencoder is to learn a latent space representation (encoding) for a data set. The general architecture is depicted in Figure 13. The input data, X, are passed to the *Encoder* network that compresses the input into smaller and meaningful representations called the *latent space representation*, Z. The *Decoder* network reconstructs the latent space representation to produce output $\hat{X}$ as similar as possible to the input [140]. Based on the specific application, the autoencoders can be built using the appropriate building blocks described in the previous chapter (i.e., CNNs, LSTM, Transformers, …).

From the point of view of the anomaly detection task, in the learning stage, only the tamper-free data are passed to the input and output of the model to learn the high-level representation of the data. In the testing stage, both tamper and tamper-free data are passed to the model, while the reconstruction error $E(X,\hat{X})$ is measured. When the reconstruction error exceeds a certain defined threshold, the data are considered to be tampered with.

A similar approach was used in [141], where the Deep-Convolutional Autoencoder (DCAE) was adopted for physical tamper attack detection and tested in indoor scenarios on custom OFDM signals. Starting from simple thresholding of the reconstruction error as the metric to judge the presence of anomalies, the method has been improved by considering the Probability Density Function (PDF) estimation of the reconstruction error. Its further extension towards multi-antenna configuration, in either a centralized or decentralized manner, has then been proposed in [142].

Although, in the above, the applications of autoencoders focused on tamper attack detection have been discussed, the autoencoder approach can also be adopted for transmitter classification [143], profiting from denoising autoencoder properties to mitigate noise interference and then improve its robustness under low SNR conditions. Again, in this paper, as well as in its predecessor [144], the principle has been evaluated on WiFi or ZigBee devices. The application to 4G device classification, among the authorized and rogue ones, has been shown in [145] on the transient part of the LTE PRACH signal with a two-dimensional wavelet coefficients graph. The autoencoder, more specifically the autoencoder with Bahdanau attention [146], has been proposed in [147] to allow physical layer authentication in highly dynamic environments, i.e., in the presence of moving users or fast-changing nearby objects. The proposed method relies on a model-based approach to predict the CSI. The decision is then based on the Mean Square Error (MSE) between the measured and predicted CSI. The use of the deep autoencoder to secure 5G-IoT devices is then proposed in [148].

## 7. Location-Based Techniques for Enhancing PHY Layer Security

Among the important threats to the integrity of 5G infrastructure is the proliferation of FBS—rogue devices impersonating legitimate base stations to intercept communications, launch attacks, or deceive users. Detecting and localizing these malicious entities is paramount for safeguarding the confidentiality, integrity, and availability of wireless communications in 5G systems [149,150]. With the potential for FBSs to exploit vulnerabilities and compromise network security, robust countermeasures are essential to mitigate their impact and ensure the trustworthiness of communication channels [68]. Further, with the recent growth of distributed, cooperative computing methods, such as federated learning [151], the localization of individual nodes could represent an additional layer of security [152]. In general, there exist several methods and approaches to localizing transmitting devices, such as FBS, among others, including the following: signal analysis, anomaly detection, ML and DL algorithms, and network-based approaches.

### 7.1. Signal Analysis and Anomaly Detection

Signal analysis and anomaly detection techniques can be applied to identifying FBSs by exploring the wireless communication signals exchanged between mobile devices and base stations. These methodologies leverage the distinctive characteristics and behaviors of legitimate base stations to differentiate them from malicious entities by exploiting signal strength clustering [153], or higher-order noise statistics [154]. Suhui et al. [155], suggested a method of Received Signal Strength (RSS)-based LTE base station localization with only a single mobile receiver when the path-loss exponent parameter is unknown. RF fingerprinting involves analyzing the unique radio frequency signatures emitted by legitimate base stations to create a reference database. By comparing the received signals with the reference fingerprints, anomalies indicative of FBSs can be detected. The usability of this technique for such a purpose has been studied in [156,157,158]. Utilizing the PSS as a signal strength metric to distinguish between genuine and false eNodeBs was introduced in [108].

### 7.2. Machine-Learning and Deep-Learning-Based Approaches

As mentioned in the previous section, various machine-learning and DL-based approaches are used more and more for RF fingerprinting, as well as for device localization [159]. The same trend is evident in the field of the localization of FBSs. Ref. [160] introduced a DL-based solution employing a CNN to locate cell towers. This approach utilized crowdsourced smartphone measurements and operator-side tower licensing data. The study demonstrated that the proposed classifier can effectively geolocate eNodeBs from other metropolitan areas or mobile operators. The authors of [107,161] demonstrated that Maximum Likelihood (ML)/DL-based approaches can effectively detect FBSs by utilizing various parameters measured at the PHY layer of a wireless link. In contrast, Mubasshir et al. [162] introduced an ML-based solution, named FBSDetector, which detects FBSs in cellular networks by analyzing network traces at Layer 3. This work also contains a good introduction and discussion about the challenges and importance of FBS detection. The results obtained from these studies, along with ongoing research in this field, indicate that ML/DL-based approaches hold significant potential for enhancing the accuracy of FBS detection and localization in the future.

### 7.3. Network-Assisted Methods

In order to localize base stations (either legitimate or rogue ones), network-assisted methods can also be used. In addition to the above-mentioned DL-empowered method [162], more straightforward solutions can be used, without the need to exploit machine learning. The Timing Advance (TA) parameter sent from the base station to the UE, or the Measurement Reports (MR) collected by the BS, have been considered in the literature [163]. The tri-lateration approach is widely used in such methods.

The exploitation of the TA parameter for base station location has already been studied thoroughly, starting from network simulations in [164] employing a Gaussian Mixture Filter, to practical field tests in Austria as described in [165]. As an example of network-assisted methods relying on measurement reports, [166] should be mentioned.

### 7.4. Angle of Arrival—Empowered Methods

The positioning of devices is recently at the center of interest of 3GPP, expecting the exploitation of sounding reference signals as the known patterns for localization. The thorough study of various localization techniques such as Angle of Arrival (AOA) or Time Difference of Arrival (TDoA) was provided in [167]. The application of AOA for localization in ultra-dense networks was studied in [168]. Recent work [169] has exploited the AOA as an enabler for physical layer authentication by providing resistance to impersonation (spoofing) attacks.

## 8. Behavior-Based Methods to Detect False Base Stations

In the previous two sections, the task of enhancing the PHY layer security of 5G networks has been seen from the perspective of machine/deep-learning methods and location-based techniques, or their combination. However, there are other families of techniques that do not fall into these two categories. Often, the securing of 5G networks is understood as a classification task aiming to distinguish between legitimate and rogue devices (UE or BS). In a technical report for 3GPP, [170] defines several solutions to be implemented on higher layers to protect against connection to a false BS, such as digitally signing the broadcasted system information, or identifying a false BS from active UE measurement reports. For the later case, the report also states that if a false BS copies the identity of a legitimate one, it is difficult to detect which one, as measured by the UE in the measurement report, belongs to a genuine base station and which one is false.

Such approaches belong to a relatively large family of methods able to detect illegitimate devices and comprises methods based on studying the discrepancies in the behavior between usual legitimate devices and their false counterparts. Note that, in many cases, at the final stage of such a method, the classifier, either a classical one or a machine-learning-based one, performs the final decision. In some cases, the behavior of the device under consideration is not studied by a single node, but rather in a network-assisted manner, in which several neighboring devices share their observations. Several exemplary suspicious marks of BS behavior have been sketched in dissertation [68], such as the following:

- sudden peaks in RSRP/SINR;
- changing the transmission power without coordination with the network;
- increased handover failure rate;
- moving base station.

Other misbehavior marks have been described in [171], from which we have selected the following examples:

- PCI outside the range of a given area;
- operating cells that have historically been powered-off outside working hours;
- received signal quality greater than usual in a given area;
- invalid PLMN identification.

Similarly, the method from [172] relied on a custom-designed state machine that analyzes portions of the RRC message logs exchanged between the base station and the UE, as well as the handover request history. According to the authors, the method outperforms several classical machine-learning-based classifiers, but was not compared with any deep-learning ones. Note that other examples of behavior methods can rely on the analysis of the measurement reports, such as [166], but as the gathered information is used to tri-laterate the position of the BS, we consider this method as the most location-aware one.

## 9. Future PHY Layer Security Challenges and Opportunities beyond 5G

**Figure 14:**
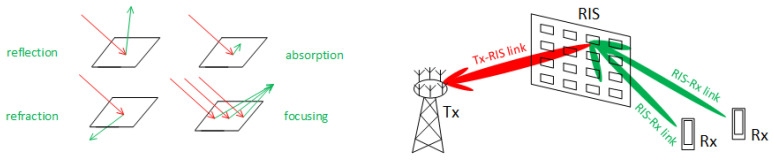
Propagation mechanisms of RIS (**left**), principle of RIS deployment (**right**).

In many countries, the deployment of 5G is still in the initial stages, with the most employed NSA mode being in the FR1 band. However, the new SA mode-based services based on private networks are currently growing, as well as the use of the FR2 band. In the near future, the promising Frequency Range 3 (FR3) band will play its role, and 5G is also expected to move to space, with the help of the current standardization activities on Non Terrestrial Networks (NTN). With the transition from 5G to 6G, several emerging technologies will gain much importance to provide additional functionalities and improve coverage. The two most prominent examples are probably Reconfigurable Intelligent Surfaces (RIS) and Joint Communication and Sensing (JCaS). The reconfigurable surfaces (see Figure 14) promise to provide coverage in areas beyond the line of sight, or to improve the signal transitions from outdoor to indoor environments, while the JCaS aims to share spectral resources, to provide environment awareness and/or to optimize the radio link performance.

With the new technologies, new risks and opportunities arise. With implicit environment awareness, the JCaS has the potential to increase PHY layer security [173], but on the other hand, it can also open up new ways of information and privacy leakage [174]. The possibility of attacking legitimate communication without any internal energy to generate jamming signals by sophisticated reflection of the signals from the legitimate transmitter to the legitimate receiver with the use of RIS has been documented in [85]. On the other hand, recently designed Simultaneous Transmitting and Receiving RIS (STAR-RIS) could provide the potential for defeating eavesdropping in a new field of so-called covert communications [175].

The current and prospective axes of PHY layer security research towards 6G thus include, but are not limited to, the means to secure JCaS transmissions [174], RIS deployments [176,177], and even to combine these technologies together [178]. In parallel, the security challenges arising from the combination of terrestrial and satellite networks [179] will also be at the center of interest with the rise of 5G NTN.

## 10. Summary

Contemporary cellular communication networks can be subject to a variety of serious security threats. To achieve fast network attack procedures, the initial phases of connection setup are not secured by the authentication mechanisms and are thus much more vulnerable to the attacks. With the use of beamforming techniques in 5G networks, users can be tracked by stealing their location information from the initial beam access reports, with increased precision due to the use of new millimeter-wave frequency bands. Thus, the potential to compromise security and privacy in the new generations of mobile communications will probably increase in the near future.

This survey paper provided an overview of 4G/5G technology from the point of view of the physical layer, and summarized the most important security threats related to the physical layer, ranging from jamming, spoofing, and message manipulation to beamforming-related attacks. We also reviewed possible countermeasures such as machine learning, location-empowered, and behavior-based techniques for the detection of false base stations—one of the key enablers of active attacks against cellular networks.

The main outputs of this survey paper provide insights into the hot topic of security threats and countermeasures at the PHY layer in 4G/5G cellular networks. This allows researcher and industry experts to identify key threats such as jamming, eavesdropping, spoofing, and more. These insights can serve as a foundation for defining and developing optimal strategies to enhance the security of 4G/5G cellular networks.

## Figures and Tables

**Table 1:** Subcarrier spacing options in 5G NR, reprinted with permissions from [56].

Subcarrier Spacing	Slots per Subframe	Meant for Carriers...	Min BW [MHz]	Max BW [MHz]
15 kHz	1	<6 GHz	4.32	49.5
30 kHz	2		8.64	99
60 kHz	4		17.28	198
120 kHz	8	>24 GHz	34.56	396
240 kHz	16		69.12	397.44

**Table 2:** Possible attack types on PHY and MAC layers [56,67].

Layer	Attack Type	Target	Effectiveness	Effects
L1 PHY	Smart Jamming	PSS	HIGH	DoS
		PBCH	MEDIUM	DoS
		SSS	MEDIUM	DoS
		PBCH DM-RS	MEDIUM	DoS
		PRACH	MEDIUM	DoS
		PDCCH	LOW	DoS
		PUCCH	LOW	DoS
	Spoofing	Implicit Beam Reporting (RA)	HIGH	User Localization
		DCI/UCI	MEDIUM	UL Jamming by UE, Resource Jamming, HARQ Failure
		PDCCH Order	MEDIUM	DoS
L2 MAC	Spoofing	BWP Switching	LOW	DoS, MITM Enabler
		CSI-RS/SP-SRS Act./Deact.	HIGH	Passive User Localization and Tracking, Massive MIMO Pilot Contamination
		SCell Act./Deact.	MEDIUM	Throughput Throttling, Battery Draining
		Timing Advance, Recommended BitRate	MEDIUM	DoS, De-synchronization
		Beam Failure Recovery	LOW	DoS

**Table 3:** Jamming spoofing parameters and J/S values for a FR1 channel bandwidth of 20 MHz and SCS of 30 KHz, reprinted with permissions from [56].

Channel/Signal	Modulation	% of REs	Sync.	Params. Required	$J/S_{\mathrm{CH}}$	$J/S_{F}$
PDSCH (DL)	{4, 16, 64, 256}-QAM	90%	No	None	0 dB	−1 dB
PBCH	QPSK	1.7%	Yes	None	0 dB	−17 dB
PDCCH	QPSK	7%	Yes	Medium	0 dB	−11 dB
PUSCH (UL)	{4, 16, 64, 256}-QAM	∼ 90%	No	None	0 dB	−1 dB
PUCCH	QPSK	∼ 10%	Yes	High	0 dB	−10 dB
PRACH	Zadoff-Chu Sequence	∼ 2%	Yes	Medium	10 dB	−7 dB
PSS (Spoofing)	M-Sequences	0.1% (3 (PSSs)	No	None	10 dB	−20 dB
SSS	Gold Sequences	0.3%	Yes	None	10 dB	−15 dB

**Table 4:** Appropriate hardware to use with OpenAirInterface and individual 4G or 5G types.

USRP	4G SISO	4G MIMO	5G NSA SISO	5G NSA MIMO	5G SA SISO	5G SA MIMO
ExpressMIMO2	yes	yes	yes	yes	yes	yes
X310	yes	yes	yes	no	yes	yes
B210	yes	yes	yes *	no	yes	yes
B200	yes	no	no	no	yes	no

* In 5G NSA mode, the 4G and 5G must operate on the same frequency due to the presence of only one shared local oscillator. Suitable for experimentation only.

**Table 5:** Appropriate hardware to use with srsRAN and individual 4G or 5G types.

USRP	4G SISO	4G MIMO	5G NSA SISO	5G NSA MIMO	5G SA SISO	5G SA MIMO
X310	yes	yes	yes	no	yes	yes
B210	yes	yes	yes *	no	yes	yes
B200	yes	no	no	no	yes	no
BladeRF	yes	yes	yes *	no	yes	yes
LimeSDR	yes	no	no	no	yes	no

* In 5G NSA mode, the 4G and 5G must operate on the same frequency due to the presence of only one shared local oscillator. Suitable for experimentation only.

**Table 6:** Examples of CNN-based machine-learning methods for 5G physical layer security enhancement.

Reference	Task	Input Data	Testbed Setup	Pre-Processing	Deep-Learning Method	Remarks
[121]	RF fingerprinting	5G PRACH Signal	USRP B210 + OAI + 6× 5G mobile phones	DCTF Extraction	CNN-based LeNet-5 structure	Experiments with Single-Channel DCTF and Multi-Channel DCTF
[122]	RF fingerprinting	LTE PRACH signal	2× USRP B205	DCTF extraction	Multi-channel CNN	-
[123]	RF fingerprinting	Wifi, LTE, 5G	USRP B210	Experiments with downsampling	VGG-style CNN	Usage of Data Augmentation
[124]	Jamming detection	5G, PSS	Not specified	PSS Correlation, DWT, EPNRE	CNN	Double threshold approach

## Data Availability

Data are contained within the article.

## Abbreviations

The following abbreviations are used in this manuscript:

3GPP	Third Generation Partnership Project
4G	Fourth Generation
5G	Fifth Generation
5GC	5G Core Network
6G	Sixth Generation
ACK	ACKnowledged
AKA	Authentication and Key Agreement
AMM	Alternating Majorization-Minimization
ARQ	Automatic Repeat reQuest
AOA	Angle of Arrival
AWGN	Additive White Gaussian Noise
BA	Beam Alignment
BCD	Block Coordinate Descent
BFR	Beam Failure Recovery
BPSK	Binary Phase Shift Keying
BS	Base Station
BWP	Bandwidth Part
CCD	Cyclic Coordinate Descent
CFI	Control Format Indicator
CFO	Carrier Frequency Offset
C/M-CCD	Conventional/Modified CCD
CORESET	COntrol REsource SET
CNN	Convolutional Neural Network
CP	Cyclic Prefix
CPE	Common Phase Error
CSI	Channel State Information
CW	Codeword
CN	Core Network
D2D	Device-to-Device
DAI	Downlink Assignment Index
DEL	Deep Learning
DCI	Downlink Control Information
DCAE	Deep-Convolutional Autoencoder
DCTF	Differential Constellation Trace Figure
DL	Downlink
DMRS	Demodulation Reference Signals
DoS	Denial of Service
DDoS	Distributed Denial of Service
DM-RS	DeModulation Reference Signals
DWT	Discrete Wavelet Transform
EPNRE	Energy Per Null Resource Elements
EPC	Evolved Packet Core
EPS	Evolved Packet System
eNB	Evolved Node B
FBS	False Base Station
gNB	gNodeB
FDD	Frequency Division Duplex
FR	Frequency Ranges
FR1	Frequency Range 1
FR2	Frequency Range 2
FR3	Frequency Range 3
GP	Guard Period
GPS	Global Positioning System
GTP	GPRS Tunnelling Protocol
GSM	Global System for Mobile communications
GT	Guard Time
HARQ	Hybrid Automatic Repeat reQuest
ICI	Inter-Carrier Interference
IMSI	International Mobile Subscriber Identity
IoT	Internet of Things
IP	Internet Protocol
IQ	In-Phase Quadrature
JCaS	Joint Communication and Sensing
LAA	License Assisted Access
LoRa	Long Range
LPWAN	Low-Power Wide Area Network
LTE	Long Term Evolution
LSTM	Long Short-Term Memory
MAC	Multiple Access Channel
MIB	Master Information Block
MIMO	Multiple-Input Multiple-Output
MITM	Man-In-The-Middle
ML	Machine Learning
ML	Maximum Likelihood
MLP	Multi-Layer Perceptron
mMIMO	massive-MIMO
MSA	Multi-head self attention
MSE	Mean Square Error
MTC	Machine Type Communication
MU-MIMO	Multi User Multiple-Input Multiple-Output
NACK	Non-ACKnowledged
NB-IoT	Narrow Band IoT
NAS	Non-Access Stratum
NLP	Natural Language Processing
NR	New Radio
NR-U	New Radio Unlicensed
NSA	Non Stand Alone
NTN	Non Terrestrial Networks
OFDM	Orthogonal Frequency Division Multiplexing
PBCH	Physical Broadcast Channel
PC-CVR	Principal Components of Channel Virtual Representation
PCFICH	Physical Control Format Indicator Channel
PCI	Physical Cell ID
PDCCH	Physical Downlink Control Channel
PO	PDCCH Order
PDSCH	Physical Downlink Shared Channel
PDF	Probability Density Function
PHY	Physical
PHICH	Physical Hybrid ARQ Indicator Channel
PRACH	Physical Random Access Channel
PSS	Primary Synchronization Signal
PT-RS	Phase Tracking Reference Signal
PUCCH	Physical Uplink Control Channel
PUSCH	Physical Uplink Shared Channel
QAM	Quadrature Amplitude Modulation
QPSK	Quadriphase Phase Shift Keying
RACH	Random Access Channel
RAN	Radio Access Network
RB	Resource Block
RE	Resource Element
RF	Radio Frequency
RIS	Reconfigurable Intelligent Surfaces
RNN	Recurrent Neural Network
RRC	Radio Resource Control
RA	Random Access
RAN	Radio Access Network
RS	Reference Signal
RNTI	Radio Network Temporary Identifier
RS	Reference Signal
RSS	Received Signal Strength
RSRP	Reference Signal Received Power
RSRQ	Reference Signal Received Quality
SA	Stand Alone
SCA	Successive Convex Approximation
SCS	Sub-Carrier Spacing
SW	Software
SDR	Software Defined Radio
SFI	Slot Format Indicator
SIB	System Information Block
SINR	Signal to Interference and Noise Ratio
SNR	Signal to Noise Ratio
SP	Semi-Presistent
SP-SRS	Single Panel Sounding Reference Signal
SR	Scheduling Request
SRS	Sounding Reference Signals
SS	Synchronization Signal
SSB	Synchronization Signal Block
SRS	Sounding Reference Signal
CSI-RS	Channel State Information Reference Signal
SSS	Secondary Synchronization Signal
STAR-RIS	Simultaneous Transmitting and Receiving RIS
STFT	Short-Time Fourier transform
TA	Timing Advance
TDD	Time Division Duplex
TD-LTE	Time Division Long Term Evolution
TDoA	Time Difference of Arrival
TPC	Transmit Power-Control
UCI	Uplink Control Information
UE	User Equipment
UL	Uplink
UMTS	Universal Mobile Telecommunications System
UP	User Plane
V2X	Vehicle-to-Everything
VLR	Visitor Location Register
WiMAX	Worldwide Interoperability for Microwave Access
WLAN	Wireless Local Area Network
CP	Control Plane
PEI	Permanent Equipment Identifier
SUPI	Subscriber Permanent Identifier
SUCI	Subscriber Concealed Identifier
ECIES	Elliptic Curve Integrated Encryption Scheme
IPsec	IP Security
ESP	Encapsulating Security Payload
IKEv2	Internet Key Exchange version 2
SBI	Service-Based Interface
TLS	Transport Layer Security
NIA	Network Integrity Algorithms
NEA	Network Encryption Algorithms
PLMN	Public Land Mobile Network
